# Supplementary material for: Machine learning-based lineage prediction from antimicrobial susceptibility testing phenotypes for Escherichia coli sequence type 131 clade C surveillance across infection types
Source: Microb Genom. 2026 Jan 19;12(1):001608. doi: 10.1099/mgen.0.001608 (PMC12816985; doi:10.1099/mgen.0.001608)
Supplement: Uncited Supplementary Material 1. [file mgen-12-01608-s001.pdf]

S1. Supplementary Figures

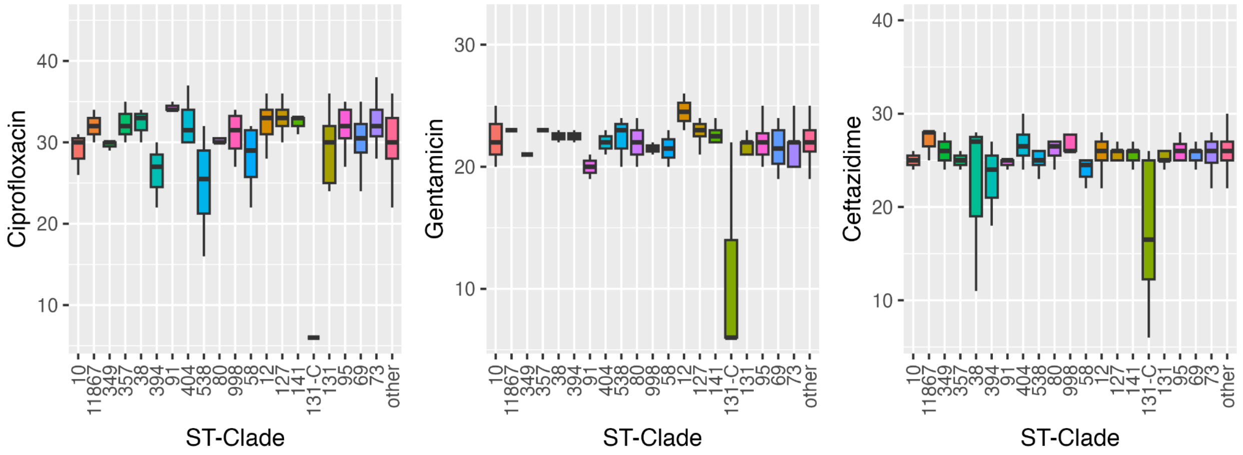

Figure S1: Boxplots for the ciprofloxacin, gentamicin, and ceftazidime zone diameter distributions in the top 20 most common population groups in the sequenced UTI dataset.

S2. Comparing ST131-C isolates in BSIs and UTIs

While a Cramer test shows that ST131-C AST distributions between BSIs and UTIs are the same, this test was conducted with a small amount of ST131-C UTI isolates. This section explores the comparison between these groups more.

Table S1: Percentage of UTI isolates in respective subgroups fall between the first and third quartile of the zone diameter distributions of the ST131-C in the genomic BSI dataset. Here, the quartiles of each zone diameter distribution were computed independently of the other two antimicrobials.

Antimicrobial	ST131-C	non ST131-C	ST394	ST58
Ciprofloxacin	100.00	2.67	0.90	0.90
Ceftazidime	42.86	83.11	64.95	48.48
Gentamicin	28.57	56.00	29.21	17.11

Observing the zone diameter distributions individually shows significant overlap of non-ST131-C UTI isolates with the zone diameter values of ST131-C found in BSIs for Ceftazidime and Gentamicin but not Ciprofloxacin (Table S1). Every ST131-C UTI fell within the first and third quartiles of at least one of the ST131-C zone diameter distributions in the BSI population (Figure S2-B). Conversely, no non-ST131 isolates from the UTI population fell within these ranges for more than two of the three antimicrobials (Figure S2-A). This lack of complete overlap in the zone diameter distributions means each of these isolates can be distinguished from ST131-C in at least one of the three antimicrobial zone diameters. This also explains why the linear logistic regression classifier failed in more cases than the nonlinear classifiers.

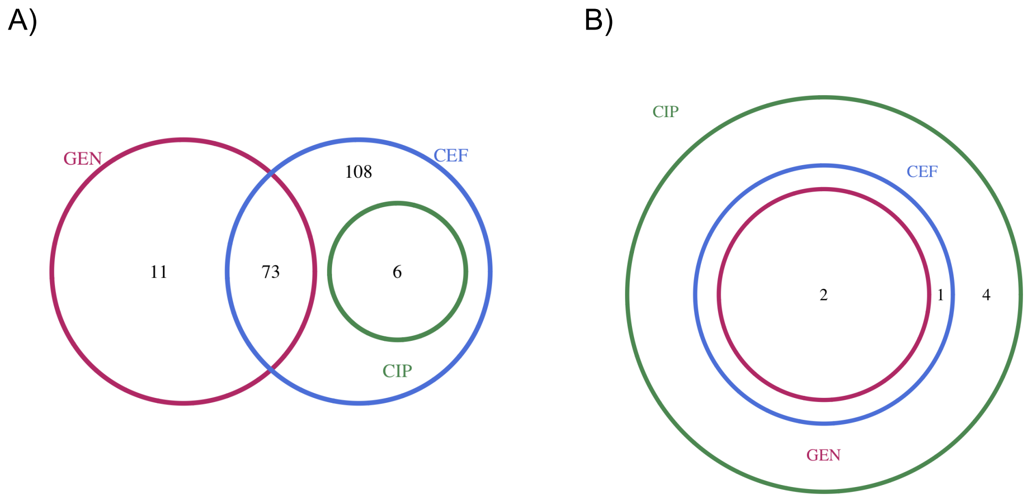

Figure S2: Venn-diagrams visualizing the isolates that fall between the first and third quartiles of the zone diameter distributions in the genomic BSI dataset from the A) non-ST131-C UTI isolates that and B) ST131-C UTI isolates.
